# Supplementary material for: Optimization of pyrolysis conditions for Catha edulis waste-based biochar production using response surface methodology
Source: Bioresour Bioprocess. 2025 Jun 17;12(1):62. doi: 10.1186/s40643-025-00866-9 (PMC12174027; doi:10.1186/s40643-025-00866-9)
Supplement: Supplementary file 1 — Supplementary material 1. [file 40643_2025_866_MOESM1_ESM.docx]

**Reference: Manuscript Number BIOB-D-24-00259R1**

**‘Optimization of Pyrolysis Conditions for *Catha edulis* Waste Based Biochar Production Using Response Surface Methodology’**

**Additional Data**

**Outputs of the Data Analysis Using R Software (Regression model and ANOVA)**

**(1) Response: Yield**

Call:

rsm(formula = y ~ FO(Temperature, Residence_Time, Particle_size) +

SO(Temperature, Residence_Time, Particle_size) - TWI(Temperature,

Residence_Time, Particle_size), data = cbind(X, y), design = "ccd")

| Estimate Std. Error t value Pr(>\|t\|) |
| --- |
| (Intercept) 1.3949e+02 3.5278e+00 39.5399 < 2.2e-16 *** |
| Temperature -3.4508e-01 1.2902e-02 -26.7468 4.515e-14 *** |
| Residence_Time -3.2882e-02 3.9553e-02 -0.8313 0.4188 |
| Particle_size -1.5801e-01 1.5089e+00 -0.1047 0.9180 |
| Temperature^2 2.7982e-04 1.2740e-05 21.9641 8.087e-13 *** |
| Residence_Time^2 -1.8350e-04 3.1850e-04 -0.5761 0.5731 |
| Particle_size^2 -8.7177e-01 5.6085e-01 -1.5544 0.1409 |
| --- |
| Signif. codes: 0 ‘***’ 0.001 ‘**’ 0.01 ‘*’ 0.05 ‘.’ 0.1 ‘ ’ 1 |
|  |
| Multiple R-squared: 0.9906, **Adjusted R-squared:** 0.9868 |
| F-statistic: 263.1 on 6 and 15 DF, **p-value**: 2.524e-14 |

**Analysis of Variance Table** (ANOVA for biochar yield)

| Response: Yield |
| --- |
| Df Sum Sq Mean Sq F value Pr(>F)  FO(Temperature, Residence Time, Particle size) 3 1391.09 463.70 363.9375 3.150e-14  PQ(Temperature, Residence Time, Particle size) 3 620.00 206.67 162.2050 1.183e-11  Residuals 15 19.11 1.27  **Lack of fit** 8 6.01 0.75 0.4013 **0.8879**  Pure error 7 13.10 1.87 |

Stationary point of response surface:

Temperature Residence_Time Particle_size

616.60496628 -89.59710101 -0.09062575

Eigenanalysis:

eigen() decomposition

$values

[1] 0.0002798240 -0.0001834981 -0.8717664380

$vectors

[,1] [,2] [,3]

Temperature 1 0 0

Residence_Time 0 1 0

Particle_size 0 0 1

**(2) Response: pH**

Near-stationary-ridge situation detected -- stationary point altered

Change 'threshold' if this is not what you intend

Call:

rsm(formula = y ~ FO(Temperature, Residence_Time, Particle_size) +

SO(Temperature, Residence_Time, Particle_size) - TWI(Temperature,

Residence_Time, Particle_size), data = cbind(X, y), design = "ccd")

| Estimate Std. Error t value Pr(>\|t\|) |
| --- |
| (Intercept) 5.5236e+00 1.0227e+00 5.4010 7.355e-05 *** |
| Temperature 9.0396e-03 3.7403e-03 2.4168 0.02886 * |
| Residence_Time 1.3029e-02 1.1467e-02 1.1363 0.27366 |
| Particle_size -4.2380e-01 4.3744e-01 -0.9688 0.34800 |
| Temperature^2 -2.2290e-06 3.6934e-06 -0.6035 0.55518 |
| Residence_Time^2 -2.4299e-05 9.2334e-05 -0.2632 0.79601 |
| Particle_size^2 4.5296e-02 1.6259e-01 0.2786 0.78436 |
| --- |
| Signif. codes: 0 ‘***’ 0.001 ‘**’ 0.01 ‘*’ 0.05 ‘.’ 0.1 ‘ ’ 1 |
|  |
| Multiple R-squared: 0.91, **Adjusted R-squared**: 0.874 |
| F-statistic: 25.29 on 6 and 15 DF, **p-value:** 4.899e-07 |
|  |

**Analysis of Variance Table** (ANOVA for biochar pH)

| Response: pH |
| --- |
| Df Sum Sq Mean Sq F value Pr(>F)  FO(Temperature, Residence Time, Particle size) 3 16.1924 5.3975 50.4054 4.554e-08  PQ(Temperature, Residence Time, Particle size) 3 0.0539 0.0180 0.1677 0.9165  Residuals 15 1.6062 0.1071  **Lack of fit**  8 0.6613 0.0827 0.6124 **0.7469**  Pure error 7 0.9449 0.1350 |

Stationary point of response surface:

Temperature Residence_Time Particle_size

0.000000 0.000000 4.678056

Eigenanalysis:

eigen() decomposition

$values

[1] 0.04529638 0.00000000 0.00000000

$vectors

[,1] [,2] [,3]

Temperature 0 1 0

Residence_Time 0 0 1

Particle_size 1 0 0

**(3) Response: FC**

Call:

rsm(formula = y ~ FO(Temperature, Residence_Time, Particle_size) +

SO(Temperature, Residence_Time, Particle_size) - TWI(Temperature,

Residence_Time, Particle_size), data = cbind(X, y), design = "ccd")

| Estimate Std. Error t value Pr(>\|t\|) |
| --- |
| (Intercept) -5.4562e+01 1.5409e+00 -35.409 7.149e-16 *** |
| Temperature 3.8054e-01 5.6354e-03 67.527 < 2.2e-16 *** |
| Residence_Time 2.0740e-01 1.7277e-02 12.005 4.299e-09 *** |
| Particle_size 1.1315e+01 6.5908e-01 17.168 2.846e-11 *** |
| Temperature^2 -3.0575e-04 5.5648e-06 -54.943 < 2.2e-16 *** |
| Residence_Time^2 -1.3524e-03 1.3912e-04 -9.721 7.242e-08 *** |
| Particle_size^2 -3.4489e+00 2.4498e-01 -14.079 4.734e-10 *** |
| --- |
| Signif. codes: 0 ‘***’ 0.001 ‘**’ 0.01 ‘*’ 0.05 ‘.’ 0.1 ‘ ’ 1 |
|  |
| Multiple R-squared: 0.9986, Adjusted R-squared: 0.998 |
| F-statistic: 1773 on 6 and 15 DF, **p-value:** < 2.2e-16 |

**Analysis of Variance Table** (ANOVA for biochar FC)

| Response: FC |
| --- |
| Df Sum Sq Mean Sq F value Pr(>F) |
| FO(Temperature, Residence_Time, Particle_size) 3 1790.09 596.70 2454.662 <2e-16 |
| PQ(Temperature, Residence_Time, Particle_size) 3 796.35 265.45 1092.002 <2e-16 |
| Residuals 15 3.65 0.24 |
| Lack of fit 8 0.83 0.10 0.259 0.9613 |
| Pure error 7 2.81 0.40 |

Stationary point of response surface:

Temperature Residence_Time Particle_size

622.317792 76.680815 1.640405

Eigenanalysis:

eigen() decomposition

$values

[1] -0.0003057465 -0.0013523764 -3.4489408261

$vectors

[,1] [,2] [,3]

Temperature 1 0 0

Residence_Time 0 1 0

Particle_size 0 0 1

**(4) Response: ASH**

Near-stationary-ridge situation detected -- stationary point altered

Change 'threshold' if this is not what you intend

Call:

rsm(formula = y ~ FO(Temperature, Residence_Time, Particle_size) +

SO(Temperature, Residence_Time, Particle_size) - TWI(Temperature,

Residence_Time, Particle_size), data = cbind(X, y), design = "ccd")

| Estimate Std. Error t value Pr(>\|t\|) |
| --- |
| (Intercept) -9.8171e+00 3.3561e-01 -29.2517 1.207e-14 *** |
| Temperature 6.5417e-02 1.2274e-03 53.2972 < 2.2e-16 *** |
| Residence_Time 3.8207e-02 3.7628e-03 10.1537 4.087e-08 *** |
| Particle_size 2.0647e+00 1.4355e-01 14.3836 3.505e-10 *** |
| Temperature^2 -4.9676e-05 1.2120e-06 -40.9867 < 2.2e-16 *** |
| Residence_Time^2 -1.9696e-04 3.0300e-05 -6.5003 1.003e-05 *** |
| Particle_size^2 -6.8768e-01 5.3356e-02 -12.8887 1.619e-09 *** |
| --- |
| Signif. codes: 0 ‘***’ 0.001 ‘**’ 0.01 ‘*’ 0.05 ‘.’ 0.1 ‘ ’ 1 |
|  |
| Multiple R-squared: 0.9983, **Adjusted R-squared**: 0.9976 |
| F-statistic: 1459 on 6 and 15 DF, **p-value**: < 2.2e-16 |

Analysis of Variance Table (ANOVA for biochar Ash)

| Response: Ash |
| --- |
| Df Sum Sq Mean Sq F value Pr(>F) |
| FO(Temperature, Residence_Time, Particle_size) 3 79.417 26.4725 2295.7190 < 2.2e-16 |
| PQ(Temperature, Residence_Time, Particle_size) 3 21.561 7.1871 623.2758 5.826e-16 |
| Residuals 15 0.173 0.0115 |
| Lack of fit 8 0.109 0.0136 1.4898 0.3063 |
| Pure error 7 0.064 0.0091 |

Stationary point of response surface:

Temperature Residence_Time Particle_size

0.000000 96.990504 1.501225

Eigenanalysis:

eigen() decomposition

$values

[1] 0.0000000000 -0.0001969606 -0.6876838225

$vectors

[,1] [,2] [,3]

Temperature 1 0 0

Residence_Time 0 1 0

Particle_size 0 0 1

**Authors’ Responses**

**Reviewer reports:
Reviewer #1:**

**Reviewer comment#****1.** The authors have **not added to the experimental sampling points on how to ensure the accuracy of the model**, and the language needs to be re-touched to ensure that it is rigorous and accurate.

**Authors’ Response:-**

Thank you for the reviewer’s concern regarding the accuracy of our models. However, we have applied a well-established methodology to design our experiment, specifically Central Composite Design (CCD) within the framework of Response Surface Methodology (RSM). This approach was used to determine the number of experimental runs as well as the combinations of selected factors (which the reviewer referred as **sampling points**), as presented in Table 1 below.

Incorporating additional points, as suggested by the reviewer, would require altering our approach and deviating from this proven methodology in favour of unestablished method(a random). It should be clear, we are confident that the selection of the operational conditions(sampling points were generated based on the CCD approach, enabled the model to capture meaningful statistical relationships. Since CCD allows for comprehensive coverage of the entire region of interest, the model generalizes effectively and is trained over a broad range. Moreover, this methodology ensures that we use a sufficiently large number of data points(**sampling points**) within the specified range, maintaining model accuracy and preventing the failure to capture key effects.

Table 1: Experimental design with the number experimental runs and
 combination of factors

| Run | Std. order | X1 | X2 | X3 | Block | Residence time | Particle size | Temp |
| --- | --- | --- | --- | --- | --- | --- | --- | --- |
| 1 | 1 | -1 | -1 | -1 | 1 | 30 | 0.5 | 350 |
| 2 | 2 | 1 | -1 | -1 | 1 | 90 | 0.5 | 350 |
| 3 | 3 | -1 | 1 | -1 | 1 | 30 | 0.5 | 650 |
| 4 | 4 | 1 | 1 | -1 | 1 | 90 | 0.5 | 650 |
| 5 | 5 | -1 | -1 | 1 | 1 | 30 | 2 | 350 |
| 6 | 6 | 1 | -1 | 1 | 1 | 90 | 2 | 350 |
| 7 | 7 | -1 | 1 | 1 | 1 | 30 | 2 | 650 |
| 8 | 8 | 1 | 1 | 1 | 1 | 90 | 2 | 650 |
| 9 | 9 | 0 | 0 | 0 | 1 | 60 | 1.25 | 500 |
| 10 | 10 | 0 | 0 | 0 | 1 | 60 | 1.25 | 500 |
| 11 | 11 | 0 | 0 | 0 | 1 | 60 | 1.25 | 500 |
| 12 | 12 | 0 | 0 | 0 | 1 | 60 | 1.25 | 500 |
| 13 | 13 | 0 | 0 | 0 | 1 | 60 | 1.25 | 500 |
| 14 | 1 | 0 | 0 | 0 | 2 | 30 | 1.25 | 500 |
| 15 | 2 | 1 | 0 | 0 | 2 | 90 | 1.25 | 500 |
| 16 | 3 | 0 | 0 | 0 | 2 | 60 | 1.25 | 350 |
| 17 | 4 | 0 | 1 | 0 | 2 | 60 | 1.25 | 650 |
| 18 | 5 | 0 | 0 | 0 | 2 | 60 | 0.5 | 500 |
| 19 | 6 | 0 | 0 | 1 | 2 | 60 | 2 | 500 |
| 20 | 7 | 0 | 0 | 0 | 2 | 60 | 1.25 | 500 |
| 21 | 8 | 0 | 0 | 0 | 2 | 60 | 1.25 | 500 |
| 22 | 9 | 0 | 0 | 0 | 2 | 60 | 1.25 | 500 |

We would like to thank the reviewer again for his/her comment we have tried to edit the language of our manuscript’s to satisfy the standards of the journal.

**Reviewer #2:** Optimization of Pyrolysis Conditions for Catha edulis Waste Based Biochar Production Using Response Surface Methodology

The article characterized the biochar produced from Catha edulis for soil amendment application. The properties investigated in the study include proximate, ultimate, BET, FTIR, and TGA, among other properties. After reading the article, my comments are enumerated as follows: **1**.      Title: Optimization of Pyrolysis Conditions for Catha edulis "Waste-Based" Biochar Production Using Response Surface Methodology

**Authors’ Response: -** Thank you for pointing out this valuable comment. In the revised manuscript it has been amended based on the reviews’ suggestion.

2.      The manuscript will benefit from careful and dedicated editorial work, including grammatical reconstruction of several sentences to better understand the data reported in the paper.

**Authors’ Response: -** We would like to thank the reviewer for his/her comment we have tried to correct the grammar and construction of sentences throughout out the manuscript’s for ease understanding the results of the paper.

3.      Separate the values of the results presented in the paper from their units. For example, page 4 line 32: "improvement(Abdulhafiz 2014; Tessfaw et al. 2020)". Add a space between "improvement" and "(". Also page 12 line 2, 350 oC, and not 350oC. There are several other similar errors within the paper. Kindly correct them.

**Authors’ Response: -**

We would like to thank the reviewer for his/her valuable comment we have separated the values of the results from corresponding units of measurement throughout the manuscript. The spacing between the words in front of each citation has also been amended.

**Reviewer #4**: After thoroughly reviewing the authors' responses to the comments and the revisions made to the manuscript, I am convinced that these changes adequately address the concerns raised during the review process.

The authors have demonstrated scientific rigor and paid close attention to improving the clarity of the manuscript, which has significantly enhanced its overall quality. In its current version, the manuscript meets the necessary standards for publication in [name of the journal].

I therefore recommend its publication in this version.

**Authors’ Response: -**

We acknowledge the dedication of the reviewer who has contributed to improving our paper. Thank you very much.

Manuscript with track changes

# **Optimization** of Pyrolysis **Conditions for** *Catha edulis* Waste-Based **Biochar Production Using Response Surface Methodology**

Abdi Birhanu^1^, Abrha Mulu Hailu^12*^, Zemene Worku^1^, Israel Tessema^1^, Kenatu Angassa^1^, Solomon Tibebu^1^

**^1^**Department Environmental Engineering, College engineering, Addis Ababa Science and Technology
 University, Sustainable Energy Center of Excellence, Bioprocess and Biotechnology Center of Excellence,
 Addis Ababa, Ethiopia..

**^2^**Deparment of Chemistry, College of Natural and Computational Sciences, Aksum University, Axum, Ethiopia.
  *****Corresponding Author: Abrha Mulu Hailu (PhD), E-mail: [chem.mulu@gmail.com](mailto:chem.mulu@gmail.com); [abrha.mulu@aau.edu.et](mailto:abrha.mulu@aau.edu.et);
 [abrha.mulu@aastu.edu.et](mailto:abrha.mulu@aastu.edu.et)

**Abstract**

*Catha edulis* (Khat) waste (KW) is one of the challenging waste managements in Ethiopian urban areas. While biochar from other biomass sources has been studied, the effect of pyrolysis conditions on *Catha edulis* waste-based biochar yield and quality remains unexplored. Therefore, this study aims to optimize the biochar production process from *Catha edulis* waste for high yield and desirable characteristics. The KW and biochar were characterized using FTIR, BET, proximate analysis and other key parameters. The results indicated that KW possesses favorable properties for thermochemical conversion, with low ash content (4.35 % wt. dry basis) and significant organic constituents (46.89 % cellulose, 28.53 % lignin, 19.62 % hemicellulose, 4.96 % extractives). The effect of pyrolysis process variables embracing reaction temperature, reaction time, and particle size on biochar yield and quality was optimized using response surface methodology (RSM) coupled with central composite design (CCD). The biochar was desirably characterized by a pH of 8.96, fixed carbon of 60.08 %, ash content of 10.55 %, and a yield of 45.12 % at the optimum production processes of 390 °C, 44 minutes, and 0.7 mm particle size. Moreover, the study found that pyrolysis temperature was the most influential factor across all responses (yield and quality). Consequently, the biochar (yield and quality) was significantly (p<0.05) influenced by pyrolysis temperature. In conclusion, the study inferred that KW holds substantial potential for biochar production with remarkable soil amendment characteristics.

**Keywords:-**Biochar, *Catha edulis* waste, CCD, Optimization, Pyrolysis conditions,
 RSM.

# Abbreviations

**AC:** Ash Content; **ANOVA:** Analysis of variances; **ASTM:** American Standard Testing Materials; **BET:** Brunauer-Emmett-Teller; **CCD:** Central Composite Design; **FC:** Fixed Carbon; **FTIR:** Fourier Transform Infrared; **KW:** *Catha edulis* Waste/Khat Waste; **MC:** Moisture Content; **RSM:** Response Surface Methodology; **TGA:** Thermo-Gravimetric analysis; **VM:** Volatile matter; **WHC:** Water Holding Capacity.

# 1. Introduction

*Catha edulis*, an evergreen shrub commonly grown in Ethiopia known as khat, has seen a significant rise in production over the years. For instance, its production rose from approximately 79,652 metric tons in 2001-2002 to 275,834.5 metric tons in 2014-2015 (Cochrane and O’Regan 2016; Rather et al. 2021). The increase in production is indirectly linked to a corresponding rise in *Catha edulis* (khat) solid waste generation. The leaves of *Catha edulis* (khat) are widely chewed as a stimulant in Ethiopia, as well as in other East Africa countries, and the Arab Peninsula countries. When the young leaves of khat are collected for local and export purposes in Ethiopia, most parts of the plant, such as older leaves and twigs, are trimmed and dumped as solid waste. Consequently, massive quantities of khat solid waste representing a significant portion of the plant’s biomass are dumped. This waste has been reducing the beauty of the cities and becoming the breeding ground of some rodents and disease-transmitting vectors. This, in turn, intensifies the poor solid waste management situation in the cities and towns of the country but is observed highly concerning in Addis Ababa.

Moreover, it is the main cause of the clogging of ditches, channels, and gutters which leads to floods in the city, and induces unwanted costs for the government and the community (Tessfaw et al. 2020). Converting such lignocellulosic biomass waste into beneficial products would address environmental and socioeconomic concerns. Therefore, the pyrolysis of Khat waste into useable biochar products can mitigate the environmental and social impacts of the waste. This process also presents an opportunity to improve acidified and contaminated soils like the effects observed with other biomass-based biochar reported by researchers (Dar et al. 2019; Kiros et al. 2020).

Biochar is a carbon-rich and porous product formed by subjecting organic biomass to pyrolysis temperatures ranging from 300 to 1000 °C with low or absence of oxygen (Tomczyk 2020; Puglia et al. 2023). It can serve as a beneficial soil amendment in agriculture due to its high carbon content, alkaline pH, stability, porosity, and surface area. It possesses a suitable surface area that can be employed as a soil remedial measure for both organic and inorganic soil pollutants via adsorption. It improves soil composition, and water retention capacity, facilitates nutrient uptake, and increases crop yield. Consequently, several studies consistently indicate that biochar improves soil quality and immobilization of heavy metals in plant growth (Kiros et al. 2020; Lehmann Johannes 2024). However, the performance of biochar for a specific application is largely dependent on its physiochemical properties which, in turn, are influenced by biochar pyrolysis conditions (Garcia et al. 2022; Murtaza et al. 2022). Therefore, optimizing its pyrolysis conditions is crucial to satisfy its specific environmental applications. Because by altering the pyrolysis conditions of the biomass, it is possible to produce biochar with various properties tailored for specific environmental applications (Brassard and Raghavan 2017).

Previously, many studies on biochar production from various biomass sources were conducted to determine optimum pyrolysis conditions. The results of these studies showed that pyrolysis temperature, residence time, and particle size were also the main factors for biochar characteristics and yield. For instance, In the optimization of pyrolysis temperature, feed particle size, and other factors biochar derived from various other biomasses, such as potato stalks (Nawaz et al. 2024); a mixture of date stones, spent coffee grounds, and cow manure (Mariyam et al. 2024); and coconut waste (Sopandi et al. 2025) reported that temperature significantly influenced the biochar yield. Likewise, the study focused on cabbage waste, temperature was also the most influential factor on biochar yield and properties, with feedstock particle size also being a significant factor (Pradhan et al. 2022). Besides, the application of biochar produced from agricultural residues like rice husk and sugarcane bagasse was effective for soil amendment and carbon sequestration when pyrolyzed under optimum conditions (Menya 2020; Murtaza et al. 2022). Similar studies on lignocellulosic biomass coffee husks and bamboo, focus on the role of feedstock characteristics and processing parameters on biochar production and characteristics (Pradhan et al. 2022; Puglia et al. 2023). However, there is limited literature on *Catha edulis* waste lignocellulosic feedstock hence, the current study fills the gap that exists in the understanding of the pyrolysis characteristics and the properties of the biochar.

Ethiopia is experiencing rapid growth in khat export commodities and is the world’s biggest producer of khat (Kandari et al. 2014). Although Khat waste is one of the abundant solid waste biomasses in Ethiopian cities, its potential for biochar production and optimization of its pyrolysis conditions has not been studied yet. Previously, researchers studied khat waste-based bio-oil/biofuel (Moreda et al. 2024), bio-adsorbent for fluoride removal from water (Fito et al. 2019; Hyacinthe Niyitegeka, Shimelis Kebede Kassahun 2023), briquette (Abdulhafiz 2014), lactic acid (Tefara et al. 2024), biochar characterizations and its application for compost nature improvement (Abdulhafiz 2014; Tessfaw et al. 2020), and khat waste based biochar nanocomposites for methylene blue removal (Kochito et al. 2024). However, none of these previous studies on Khat (*Catha edulis*) waste-based biochar optimized its pyrolysis conditions such as the pyrolysis temperature (PT), residence time (RT), particle size of feedstock (PS) and its relationship with biochar yield and quality. Overall globally, a limited studies have been conducted on the optimization of pyrolysis operating parameters of biochar production from different biomasses but nothing from Khat solid waste. Consequently, the links between pyrolysis conditions and the biochar has not yet been established, as well as Khat waste-driven biochar yield and biochar quality such as pH, fixed carbon content(FC), and ash content (AC). Therefore, the primary aim of this study is to investigate the optimal production process for biochar derived from Khat waste and to analyze how this process affects both the yield and quality of the synthesized biochar, highlighting its potential applications.

# 2. Materials and Methods

## 2.1 Sample Collection

*Catha edulis* or Khat solid waste (KW) was used as a feedstock for biochar production. Composite KW samples were collected from three randomly selected sites within the Akaki Kality sub-city of Addis Ababa City, Ethiopia. The collected Khat waste was passed through successive processing steps, including washing with distilled water, sun-drying, and oven-drying, mechanically chopping, pulverizing, and sieving to achieve a range of particle sizes, and then pyrolysis. The whole experiment was carried out at the environmental engineering laboratory of Addis Ababa Science and Technology University, Addis Ababa, Ethiopia. The overall flow diagram from feedstock collection to pyrolysis process optimization is as depicted in Fig. 1.

**
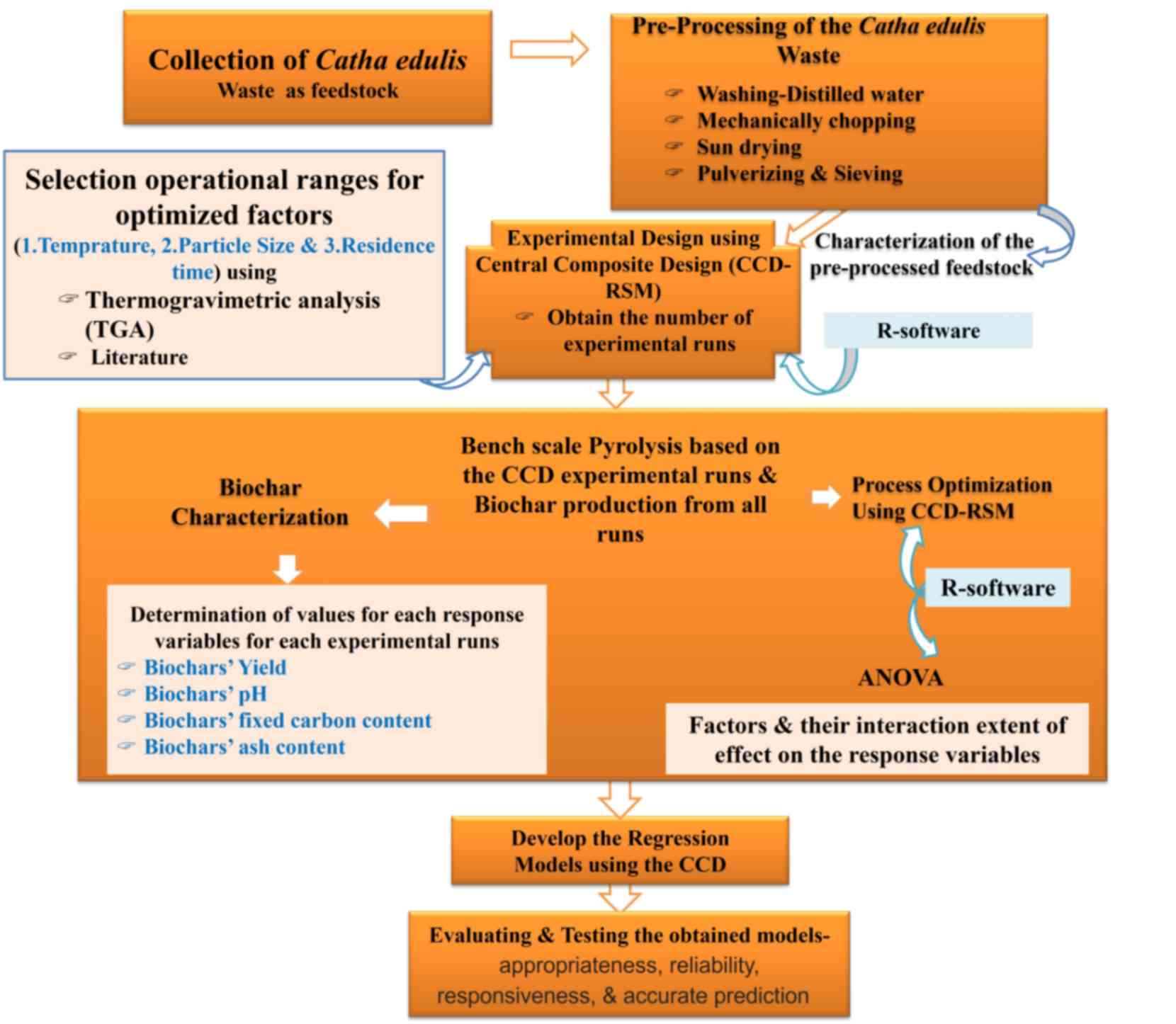
**

**Fig. 1**. Overall flow diagram from feedstock collection to pyrolysis process optimization

## 2.2 Characterization of *Catha edulis* Waste

The physicochemical characteristics of the raw feedstock are presented in Table 1. The raw waste was characterized for its moisture content (MC), volatile matter (VM), and ash content (AC) according to the standard ASTM procedures E871-82(2007), E872-82(2007), and E1755-01(2007), respectively. The fixed carbon (FC)content was obtained by subtracting the sum of MC, AC, and VM from 100 %. Besides, the oven-dried khat samples were measured for their bulk and apparent densities using ASTM procedures D 6683(2013) and 854(2013), respectively. The lignocellulosic composition of Khat waste, including hemicellulose, cellulose, lignin, and extractive was determined using the ASTM producers D1109-21, D1109-21, D1106-21, and D1107-21, respectively. The pH and EC were determined by a multi-parameter device (Geffert et al. 2019).

Thermogravimetric analysis (TGA) and Fourier-transform infrared spectroscopy (FTIR) analysis were applied to characterize biochar for selected pyrolysis conditions. The thermogravimetric analysis (TGA) of khat waste was used to study biomass breakdown patterns (weight loss) under conditions of temperature less than 700 °C and heating rate of 10 °C/min and 20 °C/min. The FTIR analysis in the range of 4000-400 cm⁻¹ was performed to identify the functional groups present in the KW using a PerkinElmer FT-IR Spectrometer, USA.

The elemental composition including carbon(C), hydrogen(H), oxygen(O), and Nitrogen(N) of the Khat biomass was estimated based on the correlation equations (1-4) for each element as described by (Shen et al. 2010).

*C= 0.635FC + 0.460VM− 0.095AC (wt%) ............................................................................*Equation 1

*H= 0.059FC + 0.060VM+ 0.010AC (wt%) ............................................................................*Equation 2

*O= 0.340FC + 0.469VM− 0.023AC (wt%)* ............................................................................Equation 3

Where FC is fixed carbon, VM is volatile matter and AC is an ash content (wt. %), and by assuming nitrogen only exists in organic and the organic component is only contributed by C, H, O, and N.

*% N = 100 -%C-%H-%O-%AC*……….......................................................................................Equation 4

The available functional groups on the surfaces of khat waste were determined using is50 ABX Fourier Transform Infrared (FTIR) in the spectrum range 4000-400cm^-1^. Thermal analysis was done using thermogravimetric at heating rates of 10 °C/min and 20 °C/min.

Table 1: Physicochemical characteristics of the feedstock

| **Parameters** | **Unit(%)** |
| --- | --- |
| VM | 65.13 ± 0.73 |
| FC | 23.43 ± 1.21 |
| AC | 4.35 ± 0.79 |
| MC | 7.08 ± 0.4 |
| Cellulose | 46.89 ± 0.64 |
| Hemicellulose | 19.62 ± 0.44 |
| Lignin | 28.53 ± 0.57 |
| Extractives | 4.96 ± 1.64 |
| Cellulose | 46.89 ± 0.64 |
| C | 47.81 |
| H | 5.74 |
| O | 41.34 |
| N | 0.75 |
| Bulk density | 0.42 ± 0.017 g/cm^3^ |
| Particle density | 1.24 g/cm^3^ |
| EC | 244 mS/cm |
| pH | 5.9 |

## 2.3 Experimental Design

In this study, Response Surface Methodology (RSM) was used to optimize the biomass pyrolysis conditions (production conditions) specifically Central Composite Design (CCD) which is a widely used experimental design approach under RSM and known for its effectiveness and adaptability. Besides, RSM allows for the identification of optimal pyrolysis conditions with fewer experimental runs than the one-factor-at-a-time approach. It minimizes the number of runs required by considering the selected factors(variables) leveraging a second-order polynomial mathematical model, including linear effects, interaction effects, and quadratic effects (El Ouadrhiri et al. 2021). Therefore, the Central Composite Design (CCD) under Response Surface Methodology (RSM) was employed to develop a mathematical model, significantly reducing the number of experimental runs compared to a full factorial design with three levels. This approach resulted in a substantial decrease in the required experiments. Further, it allowed providing data to develop response surfaces that aided in identifying the optimal pyrolysis conditions (temperature, residence time, and particle size) to maximize biochar production yield, while also ensuring desirable quality characteristics (Moussaoui et al. 2024) such as fixed carbon content, ash content, and pH. The experimental parameters for optimizing the pyrolysis process, along with their respective levels, were provided in Table 2.

To study how each factor impacts the pyrolysis process and to enhance the production of biochar through an effective and efficient pyrolysis process, the consideration of optimization of process parameters is essential to match the properties of the final biochar with its specific uses in mind. Therefore, the selection of pyrolysis process conditions or independent variables consisting of Temperature, Residence time, and Particle size is crucial due to their substantial influence on the quantity and quality of biochar produced. In this study the temperature range (between 350 °C and 650 °C) was chosen based on TGA analysis and previous studies reports (Al-Rumaihi et al. 2022; Lehmann Johannes 2024). The range was chosen between 350 °C and 650 °C to study its impact on the biochar production quality and quantity. The residence time was chosen in the range of 30 to 90 minutes to examine its effect on the carbon content and chemical stability of biochar as longer durations may aid in achieving carbonization especially, for bigger or denser feedstock. Adjusting the particle size from 0​​​ ​​mm to 2 mm also helps to assess the balance between efficiency and consistency in biochar production​​​ because smaller particles may enhance heat transfer speed while larger ones may lower energy usage and operational expenses.​​​

The optimal response of biochar was determined by examining the correlation between the responses (biochar yield, ash content, pH, and fixed carbon (FC) and the quantitative experimental variables (reaction temperature, sample particle sizes, and residence time or reaction time) (Table 2).

Table 2: Pyrolysis process optimizing parameters along with their levels

| Parameters | Low (-) | Middle (0) | High (+) |
| --- | --- | --- | --- |
| Temperature (^o^C) | 350 | 500 | 650 |
| Residence Time (min) | 30 | 60 | 90 |
| Particle size (mm) | 0.5 | 1.25 | 2 |

The primary objective of employing RSM was to efficiently determine the optimal response through regression analysis of the response surfaces while minimizing the number of experimental runs through factorial combination (Menya 2020). A minimal number of runs were obtained from the central composite design (CCD) with eight repeated central points and replicate axial points conducted using R-statistical software. CCD analysis estimates orthogonality and curvature to minimize variation in the regression coefficients. In this study the CCD output consisted of a three-level factorial design and center point experiments (0, 0), with 22 runs (Table 3). The range from the factorial point design space to the center point was considered as +1 or -1, and the range from the center to the star points was defined as alpha |α| = 1.682. The model fit was evaluated by optimizing the adjusted R^2^ and the corrected Akaike information criterion (AICc) separately. Various diagnostic and fit indices were then compared to determine the preferred model between the two. The prediction power of the model was assessed using the adjusted R^2^, and the predicted versus actual plot was examined to verify the randomness of residuals and the suitability of the model assumptions. The certainty of the model and the remaining coefficients were evaluated using the ANOVA, p-value. Notable interactions were identified by examining the magnitude of the interaction terms in the quadratic models, the shape of the surface plots, and the relative magnitude of the independent variables in perturbation plots. Optimization of the CCD-derived data was performed using the R studio optimize function, which allowed for the selection of certain parameters and their objectives (minimize, maximize, constrain in a range) based on specified weightings. The optimization function created a desirability function based on the weighting parameter and optimization goal.

Table 3: Experimental design with the number experimental runs and combination of factors

| Run | Std. order | X1 | X2 | X3 | Block | Residence time | Particle size | Temperature |
| --- | --- | --- | --- | --- | --- | --- | --- | --- |
| 1 | 1 | -1 | -1 | -1 | 1 | 30 | 0.5 | 350 |
| 2 | 2 | 1 | -1 | -1 | 1 | 90 | 0.5 | 350 |
| 3 | 3 | -1 | 1 | -1 | 1 | 30 | 0.5 | 650 |
| 4 | 4 | 1 | 1 | -1 | 1 | 90 | 0.5 | 650 |
| 5 | 5 | -1 | -1 | 1 | 1 | 30 | 2 | 350 |
| 6 | 6 | 1 | -1 | 1 | 1 | 90 | 2 | 350 |
| 7 | 7 | -1 | 1 | 1 | 1 | 30 | 2 | 650 |
| 8 | 8 | 1 | 1 | 1 | 1 | 90 | 2 | 650 |
| 9 | 9 | 0 | 0 | 0 | 1 | 60 | 1.25 | 500 |
| 10 | 10 | 0 | 0 | 0 | 1 | 60 | 1.25 | 500 |
| 11 | 11 | 0 | 0 | 0 | 1 | 60 | 1.25 | 500 |
| 12 | 12 | 0 | 0 | 0 | 1 | 60 | 1.25 | 500 |
| 13 | 13 | 0 | 0 | 0 | 1 | 60 | 1.25 | 500 |
| 14 | 1 | 0 | 0 | 0 | 2 | 30 | 1.25 | 500 |
| 15 | 2 | 1 | 0 | 0 | 2 | 90 | 1.25 | 500 |
| 16 | 3 | 0 | 0 | 0 | 2 | 60 | 1.25 | 350 |
| 17 | 4 | 0 | 1 | 0 | 2 | 60 | 1.25 | 650 |
| 18 | 5 | 0 | 0 | 0 | 2 | 60 | 0.5 | 500 |
| 19 | 6 | 0 | 0 | 1 | 2 | 60 | 2 | 500 |
| 20 | 7 | 0 | 0 | 0 | 2 | 60 | 1.25 | 500 |
| 21 | 8 | 0 | 0 | 0 | 2 | 60 | 1.25 | 500 |
| 22 | 9 | 0 | 0 | 0 | 2 | 60 | 1.25 | 500 |

## 2.4 Biochar Production (*Catha edulis* /Khat Waste Pyrolysis)

All experimental runs were carried out in a slow pyrolysis and batch-wise at the Addis Ababa Science and Technology University, environmental engineering laboratory. The prepared feedstock for an experiment was fed into the furnace in the crucible at a constant heating rate of 10°C/min since the heating rate should be kept constant to observe the effect of the three factors on biochar yield and characteristics. The pyrolysis of Khat waste was carried out at three different temperatures, three different Particle sizes, and three different residence times by controlling other parameters constant (e.g. heating rate) to examine their effect on the biochar yields.

The pyrolysis was carried out in a laboratory-scale reactor and was performed using a closed system. The reactor was a cylindrical crucible, placed inside an electrically heated furnace with a temperature control. The feedstock is monitored in an inert atmosphere, ensuring limited oxygen presence during pyrolysis. The feedstock was loaded into a crucible and pyrolyzed at different levels of temperatures, 350 °C, 500 °C, and 650 °C, residence times, 30, 60, and 90 minutes, and particle sizes, 0.5 mm, 1.25 mm, and 2 mm. The heating rate was kept constant at 10 °C/min. The reactor was allowed to cool to room temperature after pyrolysis before collecting the biochar. Finally, the biochar was then removed from the furnace, cooled in a desiccator, weighed and stored in airtight plastic containers. The biochar yield (%) from each experiment run was calculated using the formula in Equation 5**.**

Biochar 𝑌𝑖𝑒𝑙𝑑 (%) = (𝑚_Biochar_/𝑚_𝑝𝑦𝑟𝑜𝑙𝑦𝑠𝑖𝑠 𝑓𝑒𝑒𝑑𝑠𝑡𝑜𝑐𝑘_) ×100% ...................................... Equation 5

Where, 𝑚_Biochar_: mass of biochar (g) and 𝑚_pyrolysis feedstock_: mass of feedstock (g).

## 2.5 Biochar Characterization

The proximate analysis includes ash content (AC), volatile matter (VM), moisture content (MC), fixed carbon (FC), as well as particle and bulk density of the biochar samples were done based on the standard methods ASTM D1762-84, ASTM D854, and D6683 (2013), respectively. The determination of pH and EC for the biochar samples followed the same procedures as the raw Khat waste i.e. by preparing 10 % (w/w) suspension of biochar in de-ionized water and the suspension was heated to 90 °C with stirring for 20 min. The suspension was then allowed to cool to room temperature and finally the pH and the electrical conductivity were measured using multiparameter device (Tomczyk 2020; Puglia et al. 2023).

In this study, the specific surface area(SSAs) of biochar samples were measured using the Brunauer-Emmet-Teller (BET) nitrogen gas physisorption method at 77 K over the relative pressure range P/P0 = 0.05-0.30 (Zhang et al., 2017). The functional groups present in the biochar samples were analyzed using Fourier-transform infrared spectroscopy analysis (PerkinElmer FT-IR Spectrometer, USA) in the range of 4000-400 cm⁻¹. Thewater-holding capacity of biochar was determined as per the methodology stated previously (Mary et al. 2016). The percentage of water-holding capacity was calculated using Equation 5. Besides, the elemental composition (C, H, O, and N) of the biochar was done based on its proximate analysis values using the correlation Equations (6-10) proposed by (Nhuchhen 2016).

*Water holding capacity (%) =(M_2_-M_3_) /(M_3_-M_1_)*100……….………………..…….…*Equation 6

Where M_1_ is the empty container weight, M_2_ is the total weight of moist biochar within the container, and M_3_ is oven dried sample.

*C = −35.9972 + 0.7698VM + 1.3269FC + 0.3250AC (wt%)* ..........................................Equation 7

*H = 55.3678 − 0.4830VM − 0.5319FC − 0.5600AC (wt% )* ...........................................Equation 8

*O = 223.6805 − 1.7226VM− 2.2296FC − 2.2463AC (wt% )* ..........................................Equation 9

and by assuming nitrogen only exist in organic and the organic component are only contributed by C, H, O, and N.

*% N = 100 - %C - %H - %O - %AC* ......................................................................................Equation 10

The optimal temperature, particle size, and residence time of the feedstock were determined by developing mathematical models (equation 10-13) with the help of RSM, these models used a weighted optimization approach based on the overall desirability of the biochar. The weighting factors for the optimization condition selection criteria were an experimental combination with biochar characteristics FC>>Yield>>pH>>Ash according to previous similar studies (Pires de Campos 2019; Zhou et al. 2020; Moni et al. 2021; Pradhan et al. 2022).

## 2.6 Data Analysis

Designing of the experiment based on response surface methodology (RSM) and analysis of variance (ANOVA) to assess the accuracy of the models, identify significant model terms, and the effects of each independent variable and their interactions on the response variables at a 5% confidence level were done using R programming. All samples were analyzed in triplicates (n=3).

# 3. Results and Discussion

## 3.1 Khat Characteristics

The physicochemical characteristics of the raw Khat waste (*Catha edulis waste)* are detailed in Table 1 above. The proximate analysis of the Khat waste provides valuable insights into its suitability as a feedstock for biochar production. High volatile matter (VM) and fixed carbon (FC) content indicate that its potential for yielding a higher quantity of biochar on pyrolysis. Conversely, low ash content is desirable to prevent interference with the pyrolysis process and maintain the quality of the biochar. The moisture content (MC) of Khat waste, which is relatively high 43.91 ± 0.45 %, poses challenges in handling and processing, requiring additional energy for moisture removal before pyrolysis. The average VM and ash content(AC) values of the Khat waste are in agreement with previous similar study 70% and 4.35%, respectively (GEBREYES 2018). However, higher VM and lower AC compared to other biomasses like bamboo and pigeon pea stalks (Moni et al. 2021; Sahoo et al. 2021).

The lignocellulosic composition of Khat waste, including cellulose (46.89±0.64 %), hemicellulose (19.62 ± 0.44 %), lignin (28.53 ± 0.57 %), and extractive (4.96 ± 1.64 %) reveals the relative proportions of major components of the biomass. The hemicellulose content is relatively high, contributing to flexibility in the plant cell wall structure. Similarly, the lignin content is relatively high, showing greater structural integrity. The extractive content is comparatively low, consisting of non-structural components. Both the cellulose and extractive contents were found similar with other lignocellulosic biomasses (Knauf and Moniruzzaman 2004; Lehmann Johannes 2024) as there is no available data on Khat waste. The significant proportion of cellulose and lignin contents highlight the potential for biochar production and conversion.

The bulk density of Khat waste decreases as the particle size increases, with respective values of 0.433 g/cm³, 0.421 g/cm³, and 0.416 g/cm³ for particle sizes S1 (0.5mm), S2 (1.25 mm), and S3 (2 mm). This trend suggests that larger particle sizes result in lower bulk density, likely due to increased pore space. The particle density of Khat waste, averaging 1.24 g/cm³, indicates its solid density regardless of particle size. The relatively high particle density suggests compacted and solid particles. In biochar production, feedstock density influences the yield, quality, and properties of the biochar. Higher particle density indicates denser biochar, affecting porosity, surface area, and gas exchange properties important for soil amendment and carbon sequestration (Lehmann Johannes 2024). Particle size and density also affect the pyrolysis process, with smaller sizes and higher bulk densities facilitating heat transfer and improving biomass conversion, leading to higher biochar yields and improved properties.

The pH of Khat waste is slightly acidic (pH 5.9) similar to wood feedstocks (Knauf and Moniruzzaman 2004). Feedstock pH plays a significant role in biochar production, as it can influence the chemical and physical properties of the resulting biochar, including surface area, porosity, and nutrient content. It is important to note that the pH of the produced biochar differs from its feedstock due to pyrolysis-induced pH changes.

The ultimate analysis results elucidated that Khat waste contains 47.81 % carbon, 5.74 % hydrogen, 41.34 % oxygen, and 0.75 % nitrogen on a dry weight basis. Compared to other types of biomasses, Khat waste has a relatively high oxygen content. For example, wood typically has an oxygen content of around 40 %, and the high carbon content of Khat waste makes it a potential feedstock for biochar production (Major et al. 2010).

The thermogravimetric analysis (TGA) of khat revealed the biomass breakdown patterns in a controlled environment was as depicted in Fig. 2b. At the onset of the process before 100 °C is reached, a decrease of 5.74 % in weight is attributed to the evaporation of free moisture consistent with the previously identified moisture content. When the temperature increases to 220 °C a secondary weight loss phase occurs due to the evaporation of bound moisture and the beginning of organic decomposition, with most of the weight loss attributed to the release of volatile compounds, which make up 62.8 % of the waste. A substantial decrease in weight happens between 220 °C and 400 °C, which usually marks the stage where carbonization takes place. At this point in the process, the structural elements like hemicellulose, cellulose, and lignin start to break down due to heat resulting in a decrease in mass. This phase is crucial for understanding the thermal stability and decomposition kinetics of khat waste, highlighting its potential for various uses that require thermal processing, such as biochar production or energy generation. The findings from the TGA analysis not only confirm what was reported in the previous studies(Abdulhafiz 2014; Afessa et al. 2022) but also establish a clear quantitative framework for understanding the composition of khat waste. It revealed a fixed carbon content of 27.62 % and ash content of 3.85 %.

The FTIR analysis of the khat waste was done using an is50 ABX Fourier Transform Infrared (FTIR) spectrum and revealed distinct functional groups as displayed in Fig. 2a. The observed peaks indicate the presence of hydroxyl (-OH) groups, aliphatic hydrocarbons (C-H), carbonyl groups (C=O), and various oxygen-containing functional groups (C-O). These functional groups are commonly found in lignocellulosic materials, suggesting a significant presence of lignocellulosic compounds in the raw material. The FTIR analysis results are consistent with findings from previous studies that reported similar peaks in the infrared spectrum of Khat waste(Fito et al. 2019; Afessa et al. 2022).


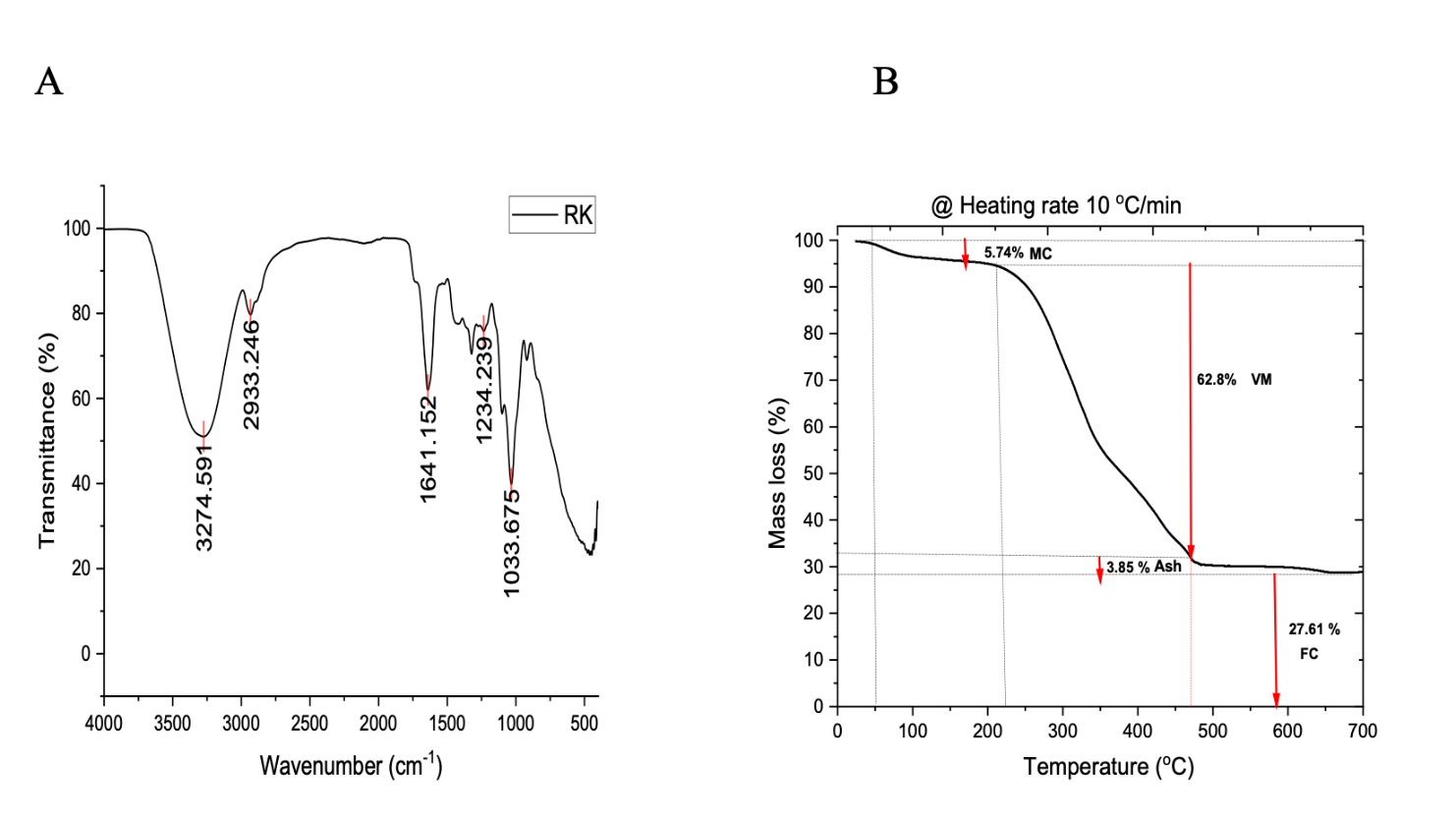


(a)

(b)

**Fig. 2**. (a)Analysis of the FTIR Spectra and (b)TGA analysis graph of Raw Khat Waste

## 3.2 Biochar Characteristics

The proximate analysis of the produced biochar includes fixed carbon (FC), volatile matter (VM), moisture content (MC), and ash content (AC) expressed as a percentage of its total weight is presented in Table 4. This analysis provides insights into the quality of the biochar*.* A lower pyrolysis temperature of 350 °C results in higher volatile matter due to incomplete carbonization and the presence of functional groups such as C=O and C-H identified by the FTIR analysis. AC represents the inorganic mineral content in biochar, which can vary across different production conditions. The range of ash content observed in this study is from 6.34 to 14.78 %. The FC content increased from 37.33 to 80.21 % across the different production conditions. Higher fixed carbon values indicate greater carbon stability, potential for carbon sequestration, and improved storage and handling characteristics of the biochar. The fixed carbon content obtained in this study is comparable to previous study (Narzari et al. 2015).

The pH values of *Catha edulis* based biochar vary from 7.92 to 11.53 across different production conditions. This range is consistent with previous finding, a pH of 8.9 at a temperature of 350 °C (Tessfaw et al. 2021). The pH levels of biochar are highly correlated with the formation of carbonates and the presence of inorganic alkalis, which are the main reasons for the alkaline characteristics of the biochar. Because during pyrolysis as the temperature rises, the total amount of base cations and carbonates also increases, pushing the pH of biochar to a range between 6.5 and 10.8. This rise in pH at higher temperatures is also connected to increased ash content and the formation of oxygen-containing functional groups during the pyrolysis process. The pH values of the Khat waste biochar in this study were alkaline, with a slight increase as the pyrolysis temperature rose. Alkaline pH is typical for biochar, and it can have positive effects on soil properties, including increased pH, enhanced nutrient availability, and improved microbial activity. The electrical conductivity (EC) of the biochar ranged from 428 to 1824 μS/cm, which is below the maximum limit (4.0 dS/m) set by Greek standards for agricultural use (Mary et al. 2016). The low EC value indicates a low level of water-soluble salts, aligning with previous findings of biochar produced from the Khat stem at a temperature of 350 °C, which reported an EC of 1.6 dS/m (Tessfaw et al. 2021).

Table 4. Characteristics of biochar produced at each experimental run or at different pyrolysis conditions

| Run | Production condition | | | Yield (%) | | Proximate analysis (%) | | | | pH | EC  (mS/cm) | Density  (g/cm^3^) | | | WHC (%) |
| --- | --- | --- | --- | --- | --- | --- | --- | --- | --- | --- | --- | --- | --- | --- | --- |
|  | **Temperature (°C )** | **Residence time (min)** | **Particle size**  **(mm)** |  |  | **MC** | **FC** | **VM** | **ASH** |  |  | **Bulk** | **Particle** | |  |
| 1 | **350** | **30** | **0.5** | **52.26** | **4.41** | | **50.72** | **36** | **8.87** | **8.41** | **894** | **0.33** | | **1.38** | **161** |
| 2 | **350** | **90** | **0.5** | **47.52** | **4.33** | | **53.64** | **32.32** | **9.71** | **9.06** | **923** | **0.30** | | **1.39** | **174** |
| 3 | **650** | **30** | **0.5** | **32.01** | **4.32** | | **74.07** | **8.1** | **13.51** | **10.5** | **1335** | **0.26** | | **1.44** | **211** |
| 4 | **650** | **90** | **0.5** | **29.17** | **4.38** | | **75.81** | **5.46** | **14.35** | **10.93** | **1394** | **0.25** | | **1.46** | **227** |
| 5 | **350** | **30** | **2** | **47.33** | **4.18** | | **54.89** | **31.57** | **9.36** | **7.92** | **512** | **0.29** | | **1.40** | **74** |
| 6 | **350** | **90** | **2** | **45.09** | **4.28** | | **58.05** | **27.44** | **10.23** | **8.53** | **621** | **0.28** | | **1.41** | **76** |
| 7 | **650** | **30** | **2** | **29.03** | **3.97** | | **77.13** | **4.95** | **13.95** | **10.06** | **879** | **0.29** | | **1.43** | **89** |
| 8 | **650** | **90** | **2** | **26.38** | **3.3** | | **80.21** | **1.71** | **14.78** | **10.63** | **911** | **0.27** | | **1.44** | **94** |
| 9 | **500** | **60** | **1.25** | **32.10** | **4.24** | | **75.60** | **6.64** | **13.52** | **9.57** | **1445** | **0.29** | | **1.43** | **187** |
| 10 | **500** | **60** | **1.25** | **32.47** | **4.21** | | **75.99** | **6.23** | **13.57** | **9.43** | **1426** | **0.29** | | **1.44** | **192** |
| 11 | **500** | **60** | **1.25** | **31.93** | **4.67** | | **75.52** | **6.26** | **13.55** | **9.64** | **1408** | **0.31** | | **1.42** | **189** |
| 12 | **500** | **60** | **1.25** | **32.56** | **4.33** | | **76.25** | **5.97** | **13.45** | **9.55** | **1397** | **0.29** | | **1.42** | **178** |
| 13 | **500** | **60** | **1.25** | **32.61** | **4.24** | | **75.83** | **6.17** | **13.76** | **9.67** | **1369** | **0.30** | | **1.43** | **196** |
| 14 | **500** | **30** | **1.25** | **34.87** | **4.34** | | **69.91** | **13.41** | **12.34** | **9.34** | **1012** | **0.31** | | **1.42** | **167** |
| 15 | **500** | **90** | **1.25** | **28.91** | **4.59** | | **74.42** | **7.11** | **13.88** | **10.46** | **1434** | **0.26** | | **1.44** | **180** |
| 16 | **350** | **60** | **1.25** | **67.43** | **3.7** | | **37.33** | **52.63** | **6.34** | **8.11** | **428** | **0.41** | | **1.36** | **107** |
| 17 | **500** | **60** | **1.25** | **32.90** | **4.44** | | **74.97** | **6.03** | **14.56** | **11.53** | **1824** | **0.24** | | **1.46** | **113** |
| 18 | **500** | **60** | **0.15** | **34.12** | **5.62** | | **68.43** | **13.61** | **12.34** | **10.45** | **1638** | **0.31** | | **1.40** | **216** |
| 19 | **500** | **60** | **2.51** | **27.75** | **3.29** | | **73.55** | **10.15** | **13.01** | **9.63** | **763** | **0.28** | | **1.43** | **67** |
| 20 | **500** | **60** | **1.25** | **31.99** | **4.4** | | **75.75** | **6.23** | **13.62** | **9.64** | **1368** | **0.32** | | **1.42** | **189** |
| 21 | **500** | **60** | **1.25** | **32.11** | **4.15** | | **75.76** | **6.6** | **13.49** | **9.52** | **1413** | **0.31** | | **1.43** | **186** |
| 22 | **500** | **60** | **1.25** | **36.05** | **4.2** | | **74.14** | **8.14** | **13.52** | **10.12** | **1407** | **0.31** | | **1.44** | **193** |

The density of biochar, including bulk density and particle density, can vary depending on the production conditions. In the case of the biochar produced from Khat waste, significant differences in bulk density were observed, while particle density showed insignificant changes. The bulk density ranged from 0.24 to 0.41 g/cm³, with a 70% difference between the lowest and highest values. The lowest bulk density of 0.24 g/cm³ was obtained at 650 °C a pyrolysis temperature, 1.25 mm particle size, and 60 minutes of residence time. The highest bulk density of 0.41 g/cm³ was observed at 247 °C temperature, 1.25 mm particle size, and 60 minutes residence time. Particle density ranged from 1.36 to 1.46 g/cm³, with only a 7% difference between the highest and lowest values. The lowest particle density of 1.36 g/cm³ was obtained at 247°C, 1.25mm particle size, and 60 minutes residence time. The highest particle density of 1.46 g/cm³ was observed at two different pyrolysis conditions: one with a temperature of 650°C, 1.25 mm particle size, and 60 minutes residence time, and the other was at 650 °C, 0.5 mm, and 90 minutes. These density values align with the general ranges for biochar density (Lehmann Johannes 2024) where bulk density typically falls within 0.2-0.6 g/cm³ and particle density ranges from 1.4-1.6 g/cm³. The observed density values for Khat waste biochar indicate in the expected range and contribute to understanding its physical characteristics and potential applications in soil amendment and carbon sequestration(Beesley et al. 2011).

The water-holding capacity (WHC) of biochar is influenced by the production conditions. The WHC of the Khat waste-based biochar was spanned from 67 to 227 %, indicating a three-fold difference between the lowest and highest values. These disparities resulted from factors such as temperature, residence time, and particle size contribute. The porosity and structure of biochar, which are influenced by the production conditions, directly affect its ability to retain water(Mary et al. 2016). Selecting the production conditions carefully is important to optimize the desired water retention properties of biochar(Beesley et al. 2011). By tailoring the production parameters, it is possible to produce biochar with a specific WHC that aligns with the intended application.

The ultimate analysis result indicates carbon content ranged from 59.65 to 80.39 % across the different production conditions. The lowest carbon content of 59.65 % was obtained at a production condition of 247 °C, 60 minutes, and a particle size of 1.25 mm. However, the highest carbon content of 80.39 % was observed at production conditions of a temperature of 650 °C, a residence time of 90 minutes, and a particle size of 2 mm. Regarding hydrogen content, values ranged from 1.72 to 4.67 %. The lowest hydrogen content of 1.72 % was obtained at a production condition with a temperature of 500 °C, a residence time of 60 minutes, and a particle size of 1.25 mm. Conversely, the highest hydrogen content of 4.67 % was observed at a production condition involving a temperature of 247 °C, a residence time of 60 minutes, and a particle size of 1. 25 mm. The oxygen content exhibited a wide range of values, from 1.36 to 28.32 %, following a similar trend as the carbon content. The specific production conditions influenced the oxygen content of the biochar. In terms of nitrogen content, the minimum value of 0.78 % was obtained at a production condition with a temperature of 247 °C, a residence time of 60 minutes, and a particle size of 1.25 mm. On the other hand, the maximum nitrogen content of 2.28 % was observed at the center point condition, which included a temperature of 500 °C, a residence time of 60 minutes, and a particle size of 1.25 mm, and these ultimate analysis findings are in agreement other findings (Tessfaw et al. 2020; Afessa et al. 2022).

The BET analysis results indicated that the biochar exhibited a substantial specific surface area (SSA). However, production conditions such as temperature, residence time, and particle size significantly influence the SSA of biochar. In this study, biochar produced at high temperatures demonstrated the highest SSA. For instance, biochar produced at 650 °C with a residence time of 30 minutes and a particle size of 0.5 mm achieved an SSA of 221.57 m²/g. In contrast, biochar produced at 500 °C and 350 °C under the same conditions showed lower SSAs of 98.15 m²/g and 57.78 m²/g, respectively. Analyzing the individual effects of temperature, residence time, and particle size, it was observed that higher temperatures generally resulted in higher SSAs. The specific area of biochar increases with temperature and residence time while decreasing with particle size (Narzari et al. 2015).

The FTIR analysis (Fig. 3) of the selected biochar samples at different pyrolysis conditions (represented by S1, S2, S3, S4, S5) reveals valuable insights into their chemical structure and the effects of pyrolysis conditions on their functional group composition. Among the factors considered, pyrolysis temperature appears to have the most significant influence on the biochar's carbon structure. The observed peaks at 3400, 2900, 1720, 1600, and 1200 cm^-1^ correspond to various vibrational modes indicative of the presence of diverse functional groups on the surface of the biochar samples. By comparing the FTIR spectra, it is evident that the evolution of these spectra is similar across all the sampled pyrolysis conditions. In terms of pyrolysis temperature, increasing it from 350 °C to 650 °C leads to several noteworthy changes. First, there is a decrease in the intensity of the O-H and C=O stretching vibrations. This can be attributed to the process of dehydration and decarboxylation occurring during pyrolysis, resulting in a reduction of these functional groups.

Likewise, the stretching and deformation vibrations of aliphatic CH_3_ and CH_2_ groups, observed, respectively, also decrease with increasing pyrolysis temperature within the range of 350 °C to 650 °C. This suggests that the aliphatic structures undergo transformation and condensation, ultimately leading to the formation of aromatic structures (Zheng et al. 2014; Lehmann Johannes 2024). Specifically, the biochar produced at 350 °C exhibits a strong aromatic C-C stretching vibration at 1600 cm^-1^, indicating the presence of aromatic structures. However, as the pyrolysis temperature increases, the intensity of this peak decreases. This observation suggests the formation of highly condensed aromatic structures in the biochars produced at higher temperatures. In summary, the FTIR analysis of the selected biochars indicates that pyrolysis temperature plays a significant role in determining their carbon structure. Higher temperatures lead to the formation of condensed aromatic structures, accompanied by the reduction of functional groups such as OH, C=O, and aliphatic CH_3_/CH_2_ groups. The particle size and residence time parameters may have additional effects on the physical properties of the biochar, which can further influence their FTIR spectra (Lehmann et al. 2021).

**Fig. 3.** FTIR spectrum of biochar derived from Khat waste at various pyrolysis conditions

## 3.3 Analysis of the Influence of Pyrolysis conditions on the Biochar Product Yield and Quality using RSM

The analysis focused on evaluating the yields and quality of biochar obtained at different pyrolysis conditions, using a Response Surface Methodology (RSM). The three pyrolysis factors considered for the current study were temperature, residence time, and particle size. The results indicated that the yield and quality of biochar were influenced by these factors, and a comprehensive analysis is presented in the subsequent paragraphs.

### 3.3.1 Biochar Yield

The analysis revealed important insights into the effects of temperature, residence time, and particle size on biochar yield during pyrolysis of the *Catha edulis waste* (Fig. 4). Pyrolysis temperature was identified as the most influential factor that significantly (p<0.05) impacted the yield. The relationship between particle size and biomass conversion is complex, with opposite responses observed for small and large particle sizes. However, the model (*Equation 11*) does not show significant interaction effects. The coefficient estimates and t-values (ratio of coefficient/ standard error) suggest that temperature has a statistically significant negative effect on yield (Zhou et al. 2020), while residence time and particle size do not significantly (P>0.05) impact the yield variable. The quadratic effects indicate concave relationships between temperature and yield, while residence time and particle size quadratic effects are not statistically significant. The model fits the data well, explaining approximately 99.06 % of the variance in the response variable. The ANOVA analysis confirms the significance of the first order and second-order terms, while the *lack of fit* values (p > 0.05) indicated that the model adequately fits the data. Besides the analysis indicates that the relative effect of temperature was much more significant than the particle size and residence time variables. The final quadratic model did not include interaction between factors (*Equation 11*). It had an R^2^-adjusted (R^2^adj) value of 0.987 and the model ANOVA p-value of <0.05.

Yield (%) = 139.487 - 0.345T -0.0329R -0.158P + 0.0002.798T^2^ -0.000184R^2.^ -0.8717P^2^ .................Equation 11

Where, T: Temperature (°C), R: Residence time (minute), P: Particle size (mm); this designation also works for equations 11-13.


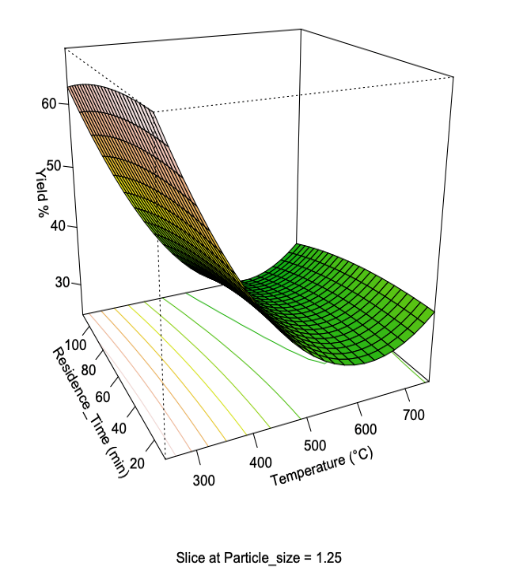


(a)


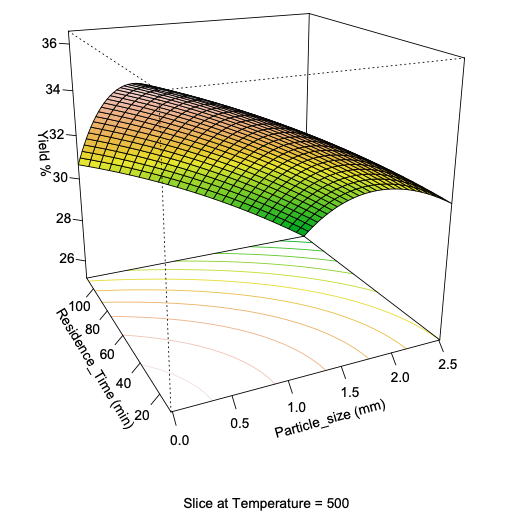


(b)


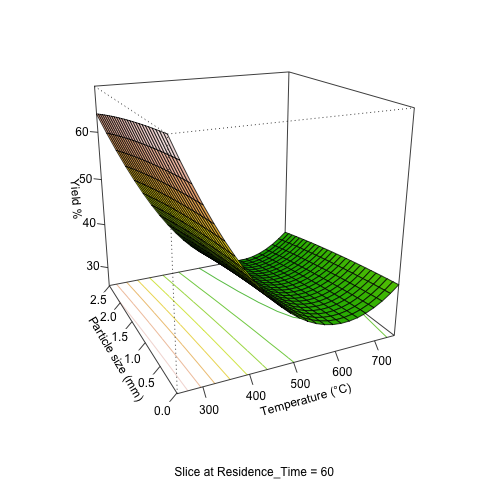


(c)

**Fig. 4.** 3-D Response surface plots influence of the interaction of pyrolysis variables on the
 biochar yield (a) temperature & residence time (b) particle size & residence time
 (c) particle size & Temperature

### 3.3.2 Biochar Quality

#### 3.3.2 .1 Biochar pH

Fig. 5 illustrates the influence of pyrolysis temperature, particle size, and residence time on the pH of the biochar. Temperature was found to have a significant (P<0.05) impact on biochar pH, while particle size and residence time had minimal effects. Higher temperatures resulted in higher pH values due to the loss of carbonaceous volatiles. The model for pH (*Equation 12)* included only the linear term for temperature, with an R^2^adj of 0.874 with a significant p-value of 4.899e-07, indicating the suitability of the model. The pH of all produced biochars was alkaline, with a maximum pH of 11.5 at 753 °C and near neutral at 250 °C. (Mukherjee et al. 2014) reported that the application of biochar at a range of pH 7 to 9 to soil preferably increases the CEC of saline soil and plant growth in comparison to biochar with higher pH, which is similar to the biochar produced at lower temperatures in this study. The combined effect of particle size and residence time on pH was minimal compared to temperature. The final model (*Equation 12)* includes only the linear term for temperature was significant (p-value <0.05). The ANOVA result confirmed the significance of the first-order terms but not the pure quadratic terms. The model had an overall fit with a Multiple R-squared of 0.91 and an Adjusted R-squared of 0.874, explaining approximately 91% of the variance in the pH response variable.

pH=5.5236+0.00904T+0.01303R-0.4238P-0.000002229T^2^ -0.0000243R^2^-0.045296P^2^.….Equation 12


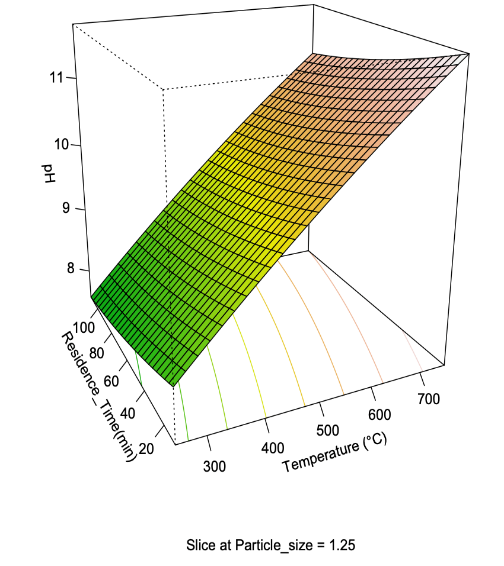


(a)


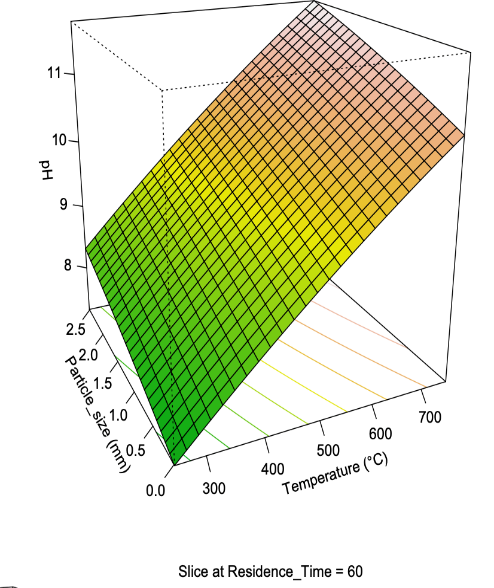


(b)


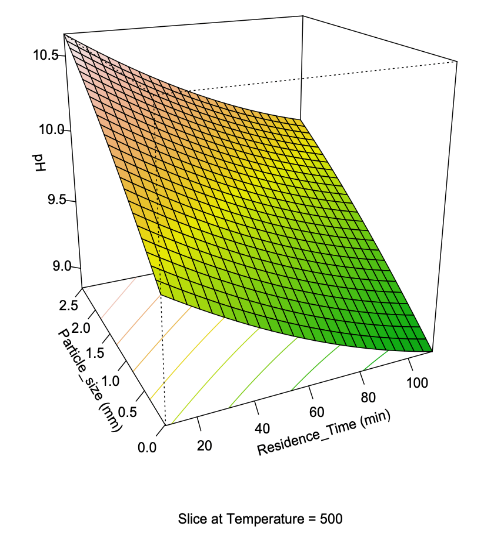


(c)

**Fig. 5**. 3-D Response surface plots influence the interaction of the pyrolysis variables on the
 biochar pH (a) pyrolysis temperature & residence time (b) particle size &
 Temperature (c) particle size & residence time

#### 3.3.2.2 Biochar Fixed carbon Content

The plots in Fig. 6 illustrate the relationship between the fixed carbon (FC) content of biochar and the pyrolysis temperature, particle size, and residence time of the feedstock. Higher pyrolysis temperatures led to increased FC content, attributed to the loss of carbonaceous volatiles. Similarly, longer residence times and larger particle sizes also resulted in higher FC content. The developed model for FC content (*Equation 13)* included both linear and quadratic terms for all factors, indicating a non-linear relationship. The model showed a high degree of fit with an adjusted R-squared value of 0.998, explaining 99.8% of the FC content variance. The ANOVA model p-value of < 0.05. The response curve (Fig. 6) depicted the combined effect of all factors on FC content, providing a visual representation of how different combinations of particle size, residence time, and temperature influenced the FC content. This result is also supported by previous findings (Pradhan et al. 2022).

FC (%) = -54.562+0.38054T+0.2074R+11.315P-0.0003058T^2^ -0.001352R^2^ -3.4489P^2^......................Equation 13


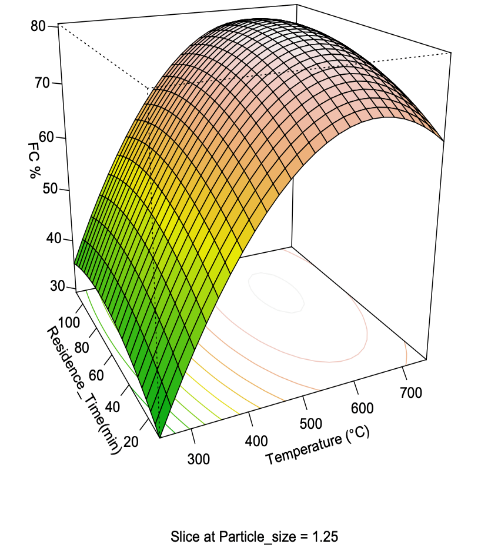


(a)


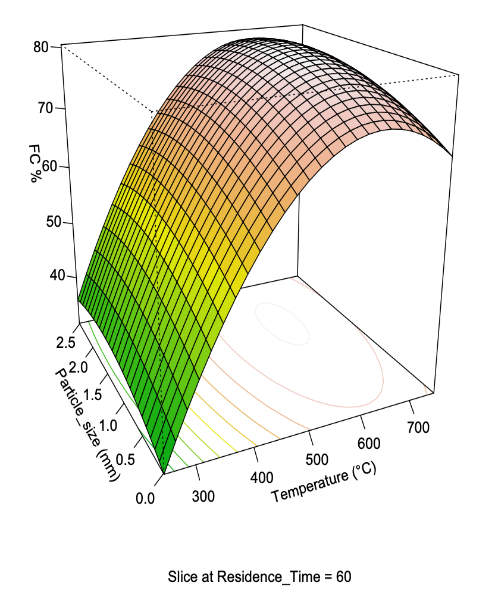


(b)

(c)

**Fig. 6.** 3-D Response surface plots influence of the interaction of pyrolysis variables on the biochar
 fixed carbon (a) temperature & residence time (b) Particle size & Temperature (c) Particle
 size &Residence

#### 3.3.2.3 Biochar Ash Content

The Fig. 7 illustrates the relationship between ash content and the pyrolysis temperature, particle size, and residence time of the feedstock. Higher pyrolysis temperatures resulted in increased ash content due to the loss of carbonaceous volatiles. Similarly, residence time and particle size had similar effects on the ash content of the biochar. The developed model for ash content (*Equation 14*) included both linear and quadratic terms for all factors, exhibiting a high degree of fit with an adjusted R-squared value of 0.9976 and a highly significant ANOVA model p-value of < 0.05. The response curve in (Fig. 7) demonstrates the combined effect of particle size and residence time on ash content. The model accounted for the overall impact of all factors. However, the Eigen analysis revealed collinearity or a near-linear relationship between the factors, suggesting redundancy or multicollinearity. In conclusion, the analysis indicated that temperature, residence time, and particle size had significant linear and quadratic effects on the ash content of the biochar.

Ash (%) = -9.8171 + 0.06542T + 0.03827R + 2.0647P -0.00004968T^2^ -0.000197R^2^ -0.6877P^2^ .........Equation 14


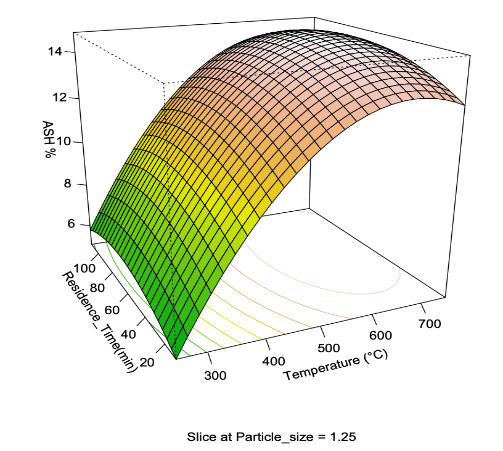


(a)


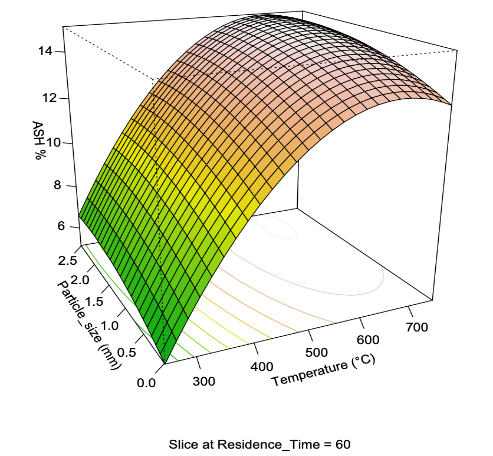


(b)


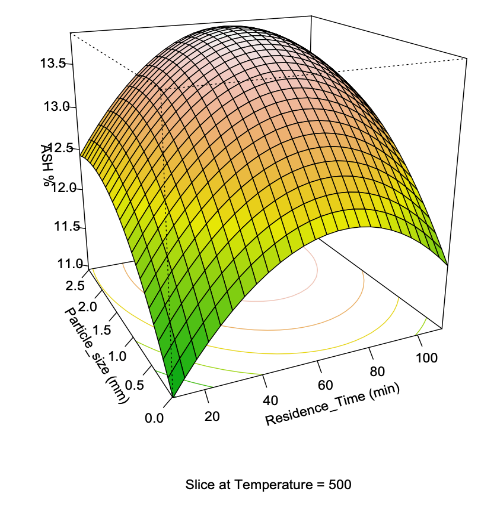


(c)

**Fig. 7.** 3-D Response surface plots influence of the interaction of pyrolysis variables on ash
 content of the biochar (a) pyrolysis temperature & residence time (b) Particle size &
 Temperature (c) Particle size & Residence

## 3.4 The Biochar Yield and Quality at Optimum Pyrolysis Conditions

Maximizing the yield of biochar production is a desirable factor to ensure the efficient utilization of feedstock resources. However, it is crucial to consider a balance between biochar yield and its quality, as excessively high yields may result in lower-quality biochar exhibits with reduced carbon content, stability, surface area, and pore structure. These factors have a significant impact on the applicability of the produced biochar such as soil improvement (Mary et al. 2016; Moni et al. 2021; Pradhan et al. 2022). Therefore, in this study, the optimal temperature, particle size, and residence time of the feedstock were determined by developing mathematical models (equation 11-14), these models used a weighted optimization approach based on the overall desirability of the biochar. The weighting factors were FC>>Yield>>pH>>Ash which has been pointed out in previous similar studies (Pires de Campos 2019; Zhou et al. 2020; Moni et al. 2021; Pradhan et al. 2022). These weighting factors were given based on the intended purpose of the biochar which is for soil amendment (agricultural purpose), where the fixed carbon (FC) was assigned the highest weight, followed by yield, pH, and ash. Based on the stated criteria, the optima biochar production conditions were found 390 °C (PT), 0.7 mm (PS), and 44 minutes (RT). At these optimal conditions, the biochar exhibited a yield of 45.12 %, a pH of 8.96, a fixed carbon content of 60.08 %, and an ash content of 10.55 %. Moreover, the result indicated that considering the balance between maximizing biochar yield and maintaining desirable quality is essential for the efficient utilization of feedstock resources. And at the optimum pyrolysis production condition.

In addition, the residuals versus fits graph with the residuals on the y-axis and the fitted values on the x-axis plots for the response variables yield, fixed carbon, ash and pH are displayed in Fig. 8, consecutively. All these residuals versus fits plots verified the assumption that the residuals are randomly distributed and have constant variance. Ideally, the points fall randomly on both sides of 0, with no recognizable patterns in the points.


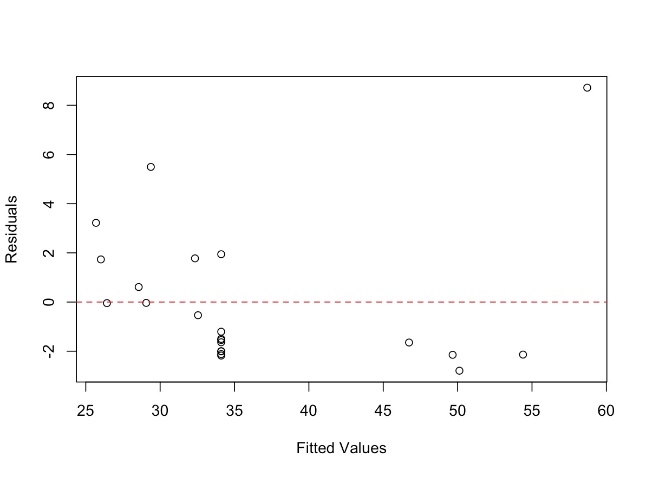

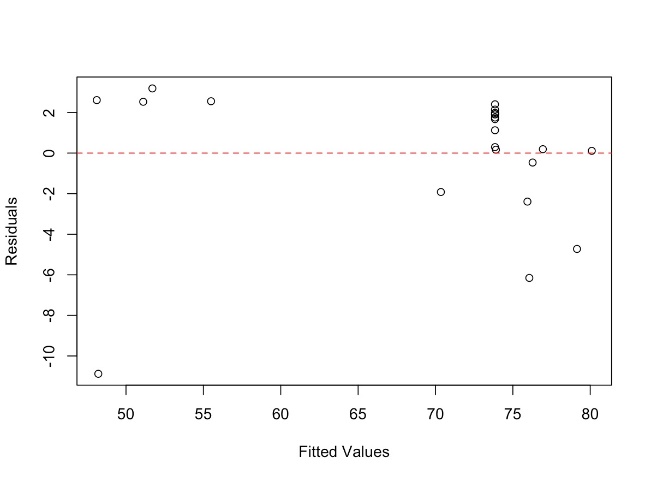

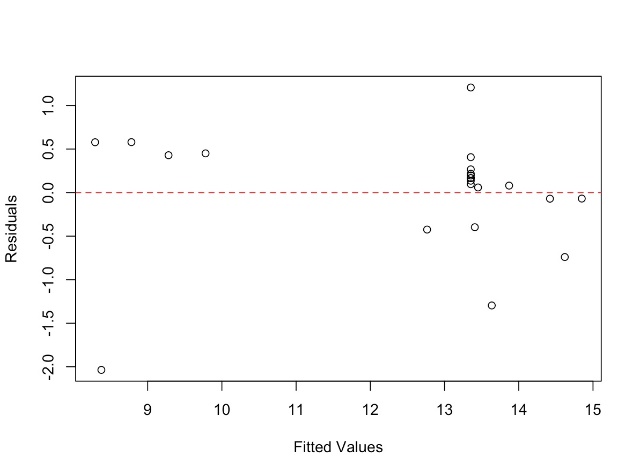

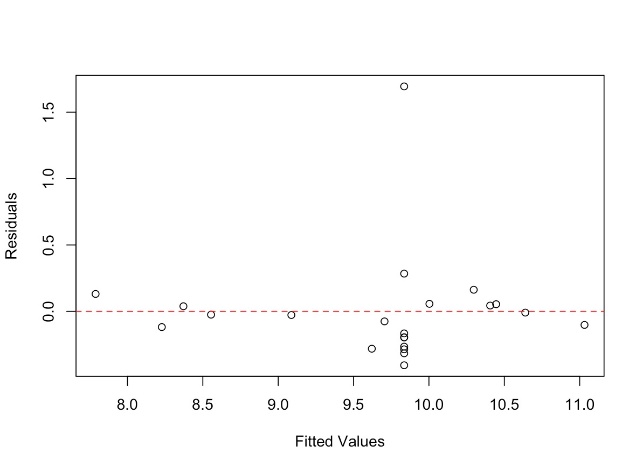


(a)

(b)

(c)

(d)

**Fig. 8.** Residual versus fits plots for Yield(a), FC(b), Ash(c), and pH(d)

# 4. Conclusion

The optimal conditions for biochar production using the RSM approach were found to be a temperature of 390 °C, reaction time of 44 min, and particle size of 0.7 mm. Under optimum values, the biochar was desirably characterized by a pH of 8.96, fixed carbon of 60.08 %, ash content of 10.55 %, and a yield of 45.12 %. While pyrolysis temperature was identified as the most influential factor (p<0.05) affecting biochar yield and quality, the roles of residence time and particle size were also notable. Longer residence times improved the fixed carbon content, while smaller particle sizes enhanced heat transfer, resulting in biochar with more uniform properties. The study inferred that *Catha edulis* waste holds substantial potential for biochar production with remarkable characteristics for soil amendment. Before application of the product in the field, further studies should be conducted to evaluate the stability and effectiveness of *Catha edulis*-derived biochar, particularly its impact on soil health, crop yield, and carbon sequestration. Moreover, assessing its economic feasibility is essential to determine both the environmental sustainability and economic viability of large-scale biochar production from this feedstock.

# Acknowledgment

Authors would like to thank Addis Ababa Science and Technology University for the funding and provision of essential laboratory facilities.

**Authors contribution**

A.B., A.M.H., and Z.W.**:** Conceptualization, Data Curation, Formal analysis, Methodology, Drafting the original manuscript.

I.T., K.A., & S.T. **:** Reviewing and Editing the manuscript.

**Funding**

The research did not receive any specific funding except laboratory facilities of Addis Ababa Science and Technology University.

**Availability of data and materials**

Not applicable.

**Statements and Declarations**

**Ethical approval -**This article does not contain any studies with human participants or animals
 performed by any of the authors.

**Competing interests-**The authors declare no competing interests.

# References

Abdulhafiz Y (2014) Liquid fuel production through pyrolysis of khat and plastic waste mixture. Addis Ababa University

Afessa G, Saka A, Tesfaye JL, et al (2022) Synthesis of Plant-Derived Khat Waste for Environmental Application. J Nanomater 2022:. https://doi.org/10.1155/2022/1094798

Al-Rumaihi A, Shahbaz M, Mckay G, et al (2022) A review of pyrolysis technologies and feedstock: A blending approach for plastic and biomass towards optimum biochar yield. Renew Sustain Energy Rev 167:112715. https://doi.org/10.1016/j.rser.2022.112715

Beesley L, Moreno-Jiménez E, Gomez-Eyles JL, et al (2011) A review of biochars’ potential role in the remediation, revegetation and restoration of contaminated soils. Environ Pollut 159:3269–3282. https://doi.org/10.1016/j.envpol.2011.07.023

Brassard P, Raghavan V (2017) Pyrolysis in auger reactors for biochar and bio-oil production : A review. Biosyst Eng 161:80–92. https://doi.org/10.1016/j.biosystemseng.2017.06.020

Cochrane L, O’Regan D (2016) Legal harvest and illegal trade: Trends, challenges, and options in khat production in Ethiopia. Int J Drug Policy 30:27–34. https://doi.org/10.1016/j.drugpo.2016.02.009

Dar AA, Rather MY, Manzoor J, et al (2019) Biochar : Preparation , Properties and Applications in Sustainable Agriculture Biochar : Preparation , Properties and Applications in Sustainable Agriculture. Int J Theor Appl Sci 11:29–40

El Ouadrhiri F, Elyemni M, Lahkimi A, et al (2021) Mesoporous Carbon from Optimized Date Stone Hydrochar by Catalytic Hydrothermal Carbonization Using Response Surface Methodology: Application to Dyes Adsorption. Int J Chem Eng 2021:. https://doi.org/10.1155/2021/5555406

Fito J, Said H, Feleke S, Worku A (2019) Fluoride removal from aqueous solution onto activated carbon of Catha edulis through the adsorption treatment technology. Environ Syst Res 8:1–10. https://doi.org/10.1186/s40068-019-0153-1

Garcia B, Rijo B, Lourinho G, Nobre C (2022) Biochar : Production , Applications , and Market Prospects in Portugal. Environemnts 9:1–21

GEBREYES EF (2018) PRODUCTION AND CHARACTERIZATION OF FUEL BRIQUETTE FROM KHAT (CATHA EDULIS FORSK) RESIDUE FOR DIVERSIFICATION OF HOUSEHOLD ENERGY SOURCES

Geffert A, Geffertova J, Dudiak M (2019) Direct method of measuring the pH value of wood. Forests 10:6–10. https://doi.org/10.3390/f10100852

Hyacinthe Niyitegeka, Shimelis Kebede Kassahun MJN (2023) Removal of fluoride from water using aluminum-modified activated carbon prepared from khat (Catha edulis) stems. J Environ Clean up Cost, Technol Tech 33:. https://doi.org/https://doi.org/10.1002/rem.21749

Kandari LS, Yadav HR, Thakur AK, Kandari T (2014) Chat ( Catha edulis ): a socio economic crop in Harar Region , Eastern Ethiopia. 1–9

Kiros Y, Mulu A, Leta S, Setarge Y (2020) Heliyon The effect of brewery sludge biochar on immobilization of bio-available cadmium and growth of Brassica carinata. Elsevier Ltd

Knauf MJ, Moniruzzaman M (2004) Lignocellulosic biomass processing: a perspective. Int Sugar J 106:147–150

Kochito J, Gure A, Abdissa N, et al (2024) MnOx- Coffea arabica Husk and Catha edulis Leftover Biochar Nanocomposites for Removal of Methylene Blue from Wastewater. Sci World J 2024:. https://doi.org/10.1155/2024/7585145

Lehmann J, Cowie A, Masiello CA, et al (2021) Biochar in climate change mitigation. Nat Geosci 14:883–892. https://doi.org/10.1038/s41561-021-00852-8

Lehmann Johannes and S. J (ed) (2024) Biochar for environmental management: science, technology and implementation. Taylor & Francis,

Major J, Rondon M, Molina D, et al (2010) Maize yield and nutrition during 4 years after biochar application to a Colombian savanna oxisol. Plant Soil 333:117–128. https://doi.org/10.1007/s11104-010-0327-0

Mariyam S, Alherbawi M, Pradhan S, et al (2024) Biochar yield prediction using response surface methodology: effect of fixed carbon and pyrolysis operating conditions. Biomass Convers Biorefinery 14:28879–28892. https://doi.org/10.1007/s13399-023-03825-6

Mary GS, Niveditha PSS, Mary GS (2016) Production , characterization and evaluation of biochar from pod ( Pisum sativum ) , leaf ( Brassica oleracea ) and peel ( Citrus sinensis ) wastes. Int J Recycl Org Waste Agric 5:43–53. https://doi.org/10.1007/s40093-016-0116-8

Menya E (2020) Optimization of pyrolysis conditions for char production from rice husks and its characterization as a precursor for production of activated carbon. Biomass Convers Biorefinery 10:57–72

Moni MNZ, Yusuf S, Manaf ASA, Rahman W (2021) Optimization of Slow Pyrolysis of Bamboo for Biochar Production using Taguchi ’ s L9 Orthogonal Array. E3S Web Conf 02004:1–5

Moreda GA, Tolasa SD, Teklemariyem DA (2024) Modeling and simulation of Khat waste fast pyrolysis for energy recovery. Heliyon 10:e24176. https://doi.org/10.1016/j.heliyon.2024.e24176

Moussaoui F, El Ouadrhiri F, Saleh EAM, et al (2024) Enhancing hydrochar production and proprieties from biogenic waste: Merging response surface methodology and machine learning for organic pollutant remediation. J Saudi Chem Soc 28:101920. https://doi.org/10.1016/j.jscs.2024.101920

Mukherjee A, Lal R, Zimmerman AR (2014) Effects of biochar and other amendments on the physical properties and greenhouse gas emissions of an artificially degraded soil. Sci Total Environ 487:26–36. https://doi.org/10.1016/j.scitotenv.2014.03.141

Murtaza G, Ahmed Z, Usman M (2022) Feedstock type, pyrolysis temperature and acid modification effects on physiochemical attributes of biochar and soil quality. Arab J Geosci 15:. https://doi.org/10.1007/s12517-022-09539-9

Narzari R, Bordoloi N, Chutia RS, et al (2015) Biochar : An Overview on its Production , Properties and Potential Benefits. In: Biology, Biotechnology and Sustainable Development. pp 13–29

Nawaz A, Razzak SA, Kumar P (2024) Pyrolysis parameter based optimization study using response surface methodology and machine learning for potato stalk. J Taiwan Inst Chem Eng 159:105476. https://doi.org/https://doi.org/10.1016/j.jtice.2024.105476

Nhuchhen DR (2016) Prediction of carbon , hydrogen , and oxygen compositions of raw and torrefied biomass using proximate analysis. FUEL 180:348–356. https://doi.org/10.1016/j.fuel.2016.04.058

Pires de Campos C (2019) Guidelines for small scale biochar production system to optimise carbon sequestration outcome. 1–139

Pradhan S, Shahbaz M, Abdelaal A, et al (2022) Optimization of process and properties of biochar from cabbage waste by response surface methodology. Biomass Convers Biorefinery 12:5479–5491. https://doi.org/10.1007/s13399-020-01101-5

Puglia M, Morselli N, Lumi M, et al (2023) Assessment of hemp hurd-derived biochar produced through different thermochemical processes and evaluation of its potential use as soil amendment. Heliyon 9:. https://doi.org/10.1016/j.heliyon.2023.e14698

Rather RA, Berhanu S, Abaynah L, Sultan M (2021) Prevalence of Khat (Catha edulis) Chewing and Its Determinants: A Respondent-Driven Survey from Hossana, Ethiopia. Subst Abuse Rehabil Volume 12:41–48. https://doi.org/10.2147/sar.s324711

Sahoo SS, Vijay VK, Chandra R, Kumar H (2021) Production and characterization of biochar produced from slow pyrolysis of pigeon pea stalk and bamboo. Clean Eng Technol 3:100101. https://doi.org/10.1016/j.clet.2021.100101

Shen J, Zhu S, Liu X, et al (2010) The prediction of elemental composition of biomass based on proximate analysis. Energy Convers Manag 51:983–987. https://doi.org/10.1016/j.enconman.2009.11.039

Sopandi TP, Sulianto AA, Anugroho F, et al (2025) RSM-optimized biochar production from young coconut waste (Cocos nucifera): Multivariate analysis of non-linear interactions between temperature, time, and activator concentration. Ind Crops Prod 223:120157. https://doi.org/10.1016/j.indcrop.2024.120157

Tefara SF, Begna Jiru E, G/Meskel Bairu A (2024) Optimization of fermentation condition for production of lactic acid from khat (“Catha edulis”) waste by using immobilized Lactobacillus plantarum. Biomass Convers Biorefinery 14:6637–6647. https://doi.org/10.1007/s13399-022-02797-3

Tessfaw ZA, Beyene A, Nebiyu A, et al (2021) Short term effects of Municipal Solid Waste compost , Khat- Derived Biochar and Co-composted Biochar on Soil quality and Faba bean Yield and Protein content. 1–16

Tessfaw ZA, Beyene A, Nebiyu A, Piko K (2020) Co-Composting of Khat-Derived Biochar with Municipal Solid Waste : A Sustainable Practice of Waste Management. Susainabllity 12:1–14

Tomczyk A (2020) Biochar physicochemical properties : pyrolysis temperature and feedstock kind effects. Rev Environ Sci Bio/Technology 19:191–215. https://doi.org/10.1007/s11157-020-09523-3

Zheng RL, Cai C, Liang JH, et al (2014) Florets & Rosettes: Meristem genes in maize and arabidopsis. Chemosphere 37:3269–3282. https://doi.org/10.1007/s11104-009-0050-x

Zhou R, Zhang M, Li J, Zhao W (2020) Optimization of preparation conditions for biochar derived from water hyacinth by using response surface methodology ( RSM ) and its application in Pb 2 + removal. J Environ Chem Eng 8:104198. https://doi.org/10.1016/j.jece.2020.104198
